# BRD4 Inhibitor AZD5153 Suppresses the Proliferation of Colorectal Cancer Cells and Sensitizes the Anticancer Effect of PARP Inhibitor

**DOI:** 10.7150/ijbs.34162

**Published:** 2019-07-21

**Authors:** Peng Zhang, Ruidong Li, Hua Xiao, Weizhen Liu, Xiangyu Zeng, Genchen Xie, Wenchang Yang, Liang Shi, Yuping Yin, Kaixiong Tao

**Affiliations:** 1 Department of Gastrointestinal Surgery, Union Hospital, Tongji Medical College, Huazhong University of Science and Technology, Wuhan 430022, China;; 2 Department of Gastroduodenal and Pancreatic Surgery, Hunan Cancer Hospital and the Affiliated Cancer Hospital of Xiangya School of Medicine, Central South University, No. 283 Tongzipo Road, Changsha, Hunan Province 410013, China.

**Keywords:** Colorectal cancer, BRD4, AZD5153, PARP inhibitor, targeted therapy.

## Abstract

**Background:** Bromodomain-containing protein 4(BRD4) is reported to play a vital role in the development of numerous malignant diseases, which is considered as a promising target for cancer therapy. AZD5153, a novel specific BRD4 inhibitor, showed potent anticancer effects in several cancer types, but its therapeutic potential has not been fully evaluated in colorectal cancer cells.

**Objective:** We sought to evaluate the therapeutic potential of BRD4 inhibition of by AZD5153 and its combined anticancer cancer effect with PARP inhibitor BMN673 *in vitro* and *in vivo* in colorectal cancer.

**Methods:** We analyzed The Cancer Genome Atlas (TCGA) database to investigate BRD4 expression in colorectal cancer patient. Clonogenic assays 、MTT assays and PI/Annexin V staining were used to determine the effect of AZD5153 and BMN673 and combination therapy on cell viability and apoptosis induction. Western blotting was applied to detect relevant molecules changes. Propidium iodide staining was performed to examine cell cycle distributions after monotherapy or combination therapy. Nude mice xenograft model was generated to confirm the therapeutic effect of AZD5153 and BMN673 combination *in vivo*, and IHC staining was used to detect the expression level of BRD4 and related markers in colorectal patient and xenograft.

**Results:** Analysis of TCGA database indicated that BRD4 was overexpressed in colorectal cancer patient. The clonogenic and MTT assays and PI/Annexin V staining demonstrated that AZD5153 significantly suppressed cell proliferation and induced apoptosis in colorectal cancer cells HCT116 and LoVo. Western blotting showed that AZD5153 inhibited the expression of *c-Myc* and increased expression of the apoptosis markers, cleaved caspase-3 and poly(ADP-ribose) polymerase (PARP), besides, we found that BRD4 knockdown could also inhibited cell proliferation and induced cell apoptosis. Moreover, AZD5153 inhibited the expression of Wee1 and impaired G2M cell cycle checkpoint, thus sensitized the anticancer effect of BMN673 *in vitro* and *in vivo*.

**Conclusion:** Our data revealed that AZD5153suppressed the proliferation of colorectal cancer cells and sensitized them to the anticancer effect of the PARP inhibitor BMN673 via Wee1 inhibition *in vitro* and *in vivo*. This suggested that targeting BRD4 might be a valuable strategy for colorectal cancer treatment.

## Introduction

Colorectal cancer (CRC) is the third leading cause of cancer-related morbidity worldwide, and constitutes approximately 10% of newly diagnosed cancers patients. Traditional treatment approaches, including surgery, radiotherapy, chemotherapy, or their combination, modestly improve the outcome of patients with CRC 1, 2. However, part of the patients still show unsatisfactory prognosis. Therefore, novel and potent therapeutic strategies are urgently needed. In the last few decades, targeted therapy and precision medicine have shown promising future for cancer treatment 3-5.

A series of targeted drugs have been approved by the US Food and Drug Administration (FDA) for CRC treatment, including inhibitors of the epidermal growth factor receptor (EGFR), vascular endothelial growth factor (VEGF)-A, and VEGF-B^6^, ^7^. However, these drugs still have some limitations and are not effective in all CRC patients. Therefore, it is imperative to find new therapeutic targets to improve the overall outcome of these patients 8, 9.

Epigenetic abnormalities have been reported to play an important role in carcinogenesis. Bromodomain and extra-terminal domain (BET) proteins have been recognized as master regulators of this process, and are characterized by the presence of two tandem bromodomains (BD1 and BD2), an extra-terminal domain (ET), and a C-terminal domain (CTD). Bromodomain-containing protein 4 (BRD4) is the most important member of the BET family and is deeply involved in the regulation of epigenetics^10^, ^11^. Functionally, it binds acetylated histones throughout the cell cycle viaits bromodomain and regulates the expression of target genes by recruiting different transcriptional regulators, such as* c-Myc*^3^, ^12^. In addition, BRD4 is reported to be a structural scaffold that indirectly regulates transcription by introducing the elongation factor, positive transcription elongation factor b (P-TEFb), into the transcription initiation complex and phosphorylating RNA polymerase II^13^, ^14^. Furthermore, BRD4 has been revealed to maintain epigenetic memory and regulates cell cycle progression^15^. More recently, BRD4 has been characterized as a key determinant of numerous malignant tumors, such as hepatocellular carcinoma (HCC), myeloid leukemia, and some other cancer types^16^-^21^. These findings suggest that BRD4 plays an important role in tumorigenesis and development, and targeting BRD4 might be a promising strategy for cancer therapy.

Taking into consideration the importance of BRD4 in malignant diseases, JQ1, a BRD2 and BRD4 dual inhibitor, has been designed, which competitively occupies the BRD4 pocket structure by binding to acetylated lysine and subsequently deploy transcription protein complexes and RNA polymerase, which inhibits the transcription of numerous genes. Furthermore, JQ1 exhibits inhibitory activity against several cancer cell lines including those derived from CRC 22, 23. However, dual targeting of BRD2 and BRD4 have presented some limitations, recent studies revealed their opposing biological effects in some cancer types, which BRD2 inhibition increased cancer cell invasiveness. Therefore, BRD4-specific inhibitors might be a better choice for cancer treatment. AZD5153 is a novel specific BRD4 inhibitor, which has presented potent anticancer effect in malignant cancer cells in pre-clinical trials, including multiple myeloma, ovarian cancer, glioblastoma, and breast cancer^24^-^28^. However, its therapeutic potential has not been fully studied in CRC. Therefore, we designed this project to investigate the anticancer effect of AZD5153 in CRC.

In this research, we examined the expression of BRD4 in colorectal cancer using TCGA database, which indicated BRD4 was overexpressed in colorectal cancer patients. Next, we examined the anticancer effect of AZD5153 in the human colorectal cancer cell lines. Most importantly, we found AZD5153 could significantly enhance the efficacy of PARP inhibitor via inhibiting G2M cell cycle checkpoint related molecule Wee1 in CRC cells.

## Materials and Methods

### Cell Culture and Reagents

9human colorectal cancer cell lines (HCT116, LoVo, RKO, Caco2, HT29, SW48, SW480, SW620, and DLD1) used in this study were obtained from the Cell Bank of the Chinese Academy of Science (Shanghai, China). The human normal colonic epithelial cell line FHC was purchased from the Wuhan University Type Culture Collection (Wuhan, China). HCT116, LoVo, and SW620 cells were cultured in McCoy's 5A, F12k, and L15 media, respectively, each containing 10% fetal bovine serum (FBS).RKO, Caco2, HT29, SW48, and SW480 cells were incubated in Dulbecco's modified Eagle's medium (DMEM) containing 10% FBS. DLD1 and FHC cells were incubated in Roswell Park Memorial Institute (RPMI)-1640 medium containing 10% FBS. All cell lines were incubated at 37 °C in a CO_2_ incubator, except for SW620 cells, which were incubated exposed to air.AZD5153、BMN673 and MK1775 were bought from Selleck (Shanghai, China). The anti-*c-Myc*, anti-cleaved poly(ADP-ribose) polymerase (PARP), anti-cleaved-caspase-3, anti-Wee1,anti-CDK1,anti-phospho-CDK1 (P-CDK1), anti-γ-H2AXand anti-Ki-67 antibodies were purchased from Cell Signaling Technology (Beverly, MA, USA). The anti-BRD4 antibody was from Abcam (Cambridge, MA, USA), and the anti-glyceraldehyde 3-phosphate dehydrogenase (GAPDH) antibody was from Proteintech Group, Inc. (Wuhan, Hubei, People's Republic of China).

### 3-(4,5-Dimethylthiazol-2-yl)-2,5-diphenyltetrazolium Bromide (MTT) Assay

Log phase cells were collected and the cell suspension density was adjusted. Then, 100 μL of medium containing 1000 cells/well were seeded in a 96-well plate, which was incubated at 37 °C in an atmosphere of 5% CO_2_ for 24 h, until cell monolayers were formed. For AZD5153 concentration-response experiments, the cells were divided into blank control (medium only), negative control (untreated), and AZD5153 (0.001, 0.01, 0.1, 1, and 10 μmol/L)-treated groups. For BRD4 knockdown experiments, the cells were divided into two groups, control and BRD4 knockdown. In order to verify whether AZD5153 and BMN673 have a sensitizing effect, there were three groups of HCT116 and LoVo cells (AZD5153, BMN673, and Combination). For concentrations, we selected 0, 20, 40, and 60 nM BMN673 for LoVo cells and0, 500, 1000, and 1500 nM BMN673 for HCT116 cells for 5days; AZD5153 was used at a ratio of 1:10.Finally, in order to verify whether AZD5153 works by reducing Wee1, we conducted a three-drug joint experiment. We selected the Wee1 specific inhibitor MK1775 (0.1μmol/L) to reduce Wee1 levels in HCT116 and LoVo cells, another group used DMSO as a control. We treated HCT116 with 2 μmol/L AZD5153 and 0.25μmol/L BMN673 (individually and in combination), while LoVo cells were treated with 1 μmol/L AZD5153 and 0.5μmol/L BMN673 (individually and in combination).After incubation for 72 h at 37 °C exposed to 5% CO_2_, 10 μLMTT solution (5 mg/mL) was added to each well and incubated for an additional 4 h. The culture was stopped, the medium was carefully aspirated, and 150 μL dimethyl sulfoxide (DMSO) was added to each well, followed by shaking at low speed on a shaker for 20 min to fully dissolve the formazan crystals. The absorbance of each well was measured at a wavelength of 490 nm (OD_490_) using an enzyme-linked immunosorbent assay plate reader. At least five replicates were used in each MTT experiment, which was repeated three times.

### Clonogenic Assay

Log phase cells were collected, the cell suspension density was adjusted to 1000 cells/well, and the cells were incubated at 37 °C exposed to 5% CO_2_ for 24 h. When the HCT116 cells were completely adherent, AZD5153 was added at concentrations of 0, 1, or 2 μmol/L per well. To the LoVo cells, AZD5153 was added at concentrations of 0, 0.5, and 1 μmol/L per well. In order to assess the combined activity of AZD5153 and BMN673, HCT116 and LoVo cells were divided into three treatment groups (AZD5153, BMN673, and Combination). For these combination experiments, we selected 2μmol/L AZD5153 and 0.25μmol/L BMN673 forHCT116, and1μmol/L AZD5153 and 0.5μmol/LBMN673 for LoVo. The cells were incubated at 37°C exposed to 5% CO_2_ in an incubator with saturated humidity. After cultivation for 10 days, the colonies were counted visually, with > 50 cells/colony considered a clone.

### Annexin V/ Propidium Iodide (PI) Assay for Apoptosis

HCT116 and LoVo cells were seeded in 6-well plates at a density of 2 × 105 cells/well. AZD5153, at concentrations of 0, 0.5,1, and 2μmol/L was added to the medium. Another 6-well plate was treated with small interfering RNA for BRD4 (siBRD4) to knockdown the BRD4 gene, as indicated in section 2.8. For drug treatment experiments, we treated HCT116 with 2μmol/L AZD5153 and 0.25μmol/L BMN673 (individually and in combination), while LoVo cells were treated with 1μmol/L AZD5153 and 0.5μmol/L BMN673 (individually and in combination). Finally, in order to verify whether AZD5153 works by reducing Wee1, we conducted a three-drug joint experiment. We selected the Wee1 specific inhibitor MK1775 (0.1μmol/L) to reduce Wee1 levels in HCT116 and LoVo cells. The other group used DMSO as a control. AZD5153 and BMN673 have the same concentration as above. After cultivation for 48 h, the cells were harvested, washed with phosphate-buffered saline (PBS), and stained with the Annexin V/ Propidium Iodide (PI) Apoptosis Detection Kit. The fluorescence of the cells was visualized using flow cytometry.

### Cell Cycle Analysis

The Cell Cycle and Apoptosis Assay Kit (Beyotime, Shanghai, China) was used to assess the effect ofAZD5153; AZD5153, BMN673, and the combination of the two drugs on the cell cycle of HCT116 and LoVo cells, following the manufacturer's instructions. The cells were seeded into 6-well plates at a density of 2 × 105 cells/well. First, we treated HCT116 with 2μmol/L and LoVo1μmol/L with cells with single drug AZD5153.Then we treated HCT116 with 2μmol/L AZD5153 and 0.25μmol/L BMN673 (individually and in combination), while LoVo cells were treated with 1μmol/L AZD5153 and 0.5μmol/L BMN673 (individually and in combination). The cell cycle was measured by flow cytometry 48 h after treatment.

### Western Blot Analysis

The cells seeded in 6-well plates were washed twice with pre-chilled PBS, and lysed on ice for 20 min with radioimmunoprecipitation assay (RIPA) buffer containing 1:100 phenyl methanesulfonyl fluoride (PMSF). The cell lysates were centrifuged at 4 °C at 12000 rpm for 20 min, and the supernatants (containing total soluble cellular protein) were collected. Protein concentrations in the supernatants were quantified using the bicinchoninic acid (BCA) assay. The proteins were denatured at 100 °C or 5 min after dilution with 5× protein loading buffer. The proteins were separated using sodium dodecyl sulfate-polyacrylamide gel electrophoresis (SDS-PAGE) and transferred to membranes, which were blocked for 2 h. Then, the membranes were incubated with primary antibodies (BRD4, 1:1000; cleaved-PARP, 1:1000; cleaved-caspase-3, 1:1000; *c-Myc*, 1:1000; Wee1,1:1000;CDK1,1:1000;P-CDK1,1:1000;γ-H2AX, 1:1000and GAPDH, 1:4000). After washing with Tris-buffered saline plus Tween^®^20 (TBST), the membranes were incubated with secondary antibody (1:3000) for 2 h, and chemiluminescence was used to visualize the protein bands with X-ray film. The intensity of each band was analyzed using Image J software.

### Immunohistochemistry

Paraffin-embedded tissue preparations were serially sectioned at a thickness of 4 μm and mounted on slides previously treated with polylysine. Afterward, the slides were heatedate60 °C for 2 h and then overnight at 37 °C. The sections were dewaxed in xylene for 2× 10 min, rehydrated using an alcohol gradient series (100%, 95%, 80%, and 70% ethanol), and then washed with double distilled water for 3× 5 min. Endogenous peroxidase was blocked with 3% H2O2 and the slides were washed with double distilled water for 3× 5 min. Antigen retrieval was performed by heating the tissue in a microwave oven for 10 min, cooling, and then washing in PBS for 2× 5 min. The tissue was blocked in goat serum blocking solution, which was added drop wise and incubated for 15 min at room temperature. Then, the tissue was incubated with anti-BRD4 primary antibody (1:200 dilution) overnight at 4°C and then washed in PBS for 3× 3 min. Then, the biotinylated secondary antibody was added drop wise, followed by incubation at 37 °C for 15 min and washing in PBS for 3× 3 min. Then, the tissue was incubated with horseradish peroxidase-labeled streptavidin solution at 37 °C for 15 min, and washed in PBS for 3× 3 min. This was followed by treatment with 3,3'-diaminobenzidine (DAB) dye for 5-10 min and thorough washing. The tissue was counterstained with hematoxylin, thoroughly washed, alcohol dehydrated, xylene-cleared for transparency, and mounted. Resected tumors from the mouse model were fixed in 10% formaldehyde in PBS for 24 h, immersed in 70% ethanol, and then embedded in paraffin. The primary antibodies, anti-cleaved PARP(1:200), anti-cleaved-caspase-3(1:600), and anti-Ki-67(1:400), were used for the immunohistochemistry. The immunohistochemical staining results were assigned a mean score considering both the intensity of staining and the proportion of tumor cells with an unequivocal positive reaction. Each section was independently assessed by 2 pathologists without prior knowledge of patient data. Positive reactions were defined as those showing brown signals in the cell cytoplasm. A staining index (values, 0-12) was determined by multiplying the score for staining Intensity with the score for positive area. The intensity was scored as follows: 0, negative; 1, weak; 2, moderate; and 3, strong. The frequency of positive cells was defined as follows than5%; 1, 5% to 25%:2, 26% to50%; 3, 51% to 75%; and 4, greater than 75%.When the staining was heterogeneous, we scored it as follows: each component was scored. Independently and summed for the results. For statistical analysis, scores of 0 to 7 were considered low expression and scores of 8 to 12considered high expression.

### RNA Interference

The cells were seeded in 6-well plates and cultured for 12-24 h until they reached a confluence of 30-40%, and the medium was changed to that without antibiotics. Transfection of siRNA was performed using Lipofectamine^®^ 6000. After 6 h to eliminate the toxic effects of transfection, the medium was replaced with complete medium. The cells were cultured for 48 h, harvested for protein extraction, and then apoptosis was measured using flow cytometry. The same method was used to transfect cells in a 96-well plate. The MTT assays were performed after siRNA transfection. The effects of knockdown of the *BRD4* gene on cell proliferation and apoptosis were determined.

### Animal Studies

The study was conducted under the permission of the ethics committee of Tongji Medical College, Huazhong University of Science and Technology, China. For AZD5153/BMN673 studies, 6-week-old male Balb/c/nu mice were obtained from Beijing Huafukang Biotechnology Co., Ltd. HCT116 tumor cells (2× 10^6^) in 100μL PBS were injected into the right flank of the nudemice. After the tumors reached approximately 200mm^3^, we treated them ice in cohorts as follows (n=6 per group): vehicle (20% hydroxypropyl-β-cyclodextrin, 5% DMSO, and 0.2% Tween^®^80 in saline), AZD5153 (5 mg/kg), BMN673 (0.33mg/kg), and the AZD5153/BMN673 combination (same doses as individually) via gavage for 3 weeks. The mice were examined biweekly for the effects of tumor burden and tumor growth. Tumor volume was calculated weekly from caliper measurements of the smallest diameter (SD) and largest diameter (LD) using the formula: volume=[LD × SD^2^]× π/6.Mouse body weight and tumor growth were monitored every second day using a balance and calipers until the end of the experimental period. Then, the tumors were excised and weighed. The excised tumors were formalin-fixed for immunohistochemistry.

### Statistical Analysis

The results are presented as means ± standard deviation (SD). Statistical analysis was performed using one-way analysis of variance (ANOVA) followed by *t*-tests to determine significant differences between two groups. Statistical analyses were performed using the statistical package for the social sciences (SPSS) 17.0 software and p-values < 0.05 were regarded as statistically significant.

## Results

### BRD4 is Highly Expressed in Colon Cancer

**Figure 1:**
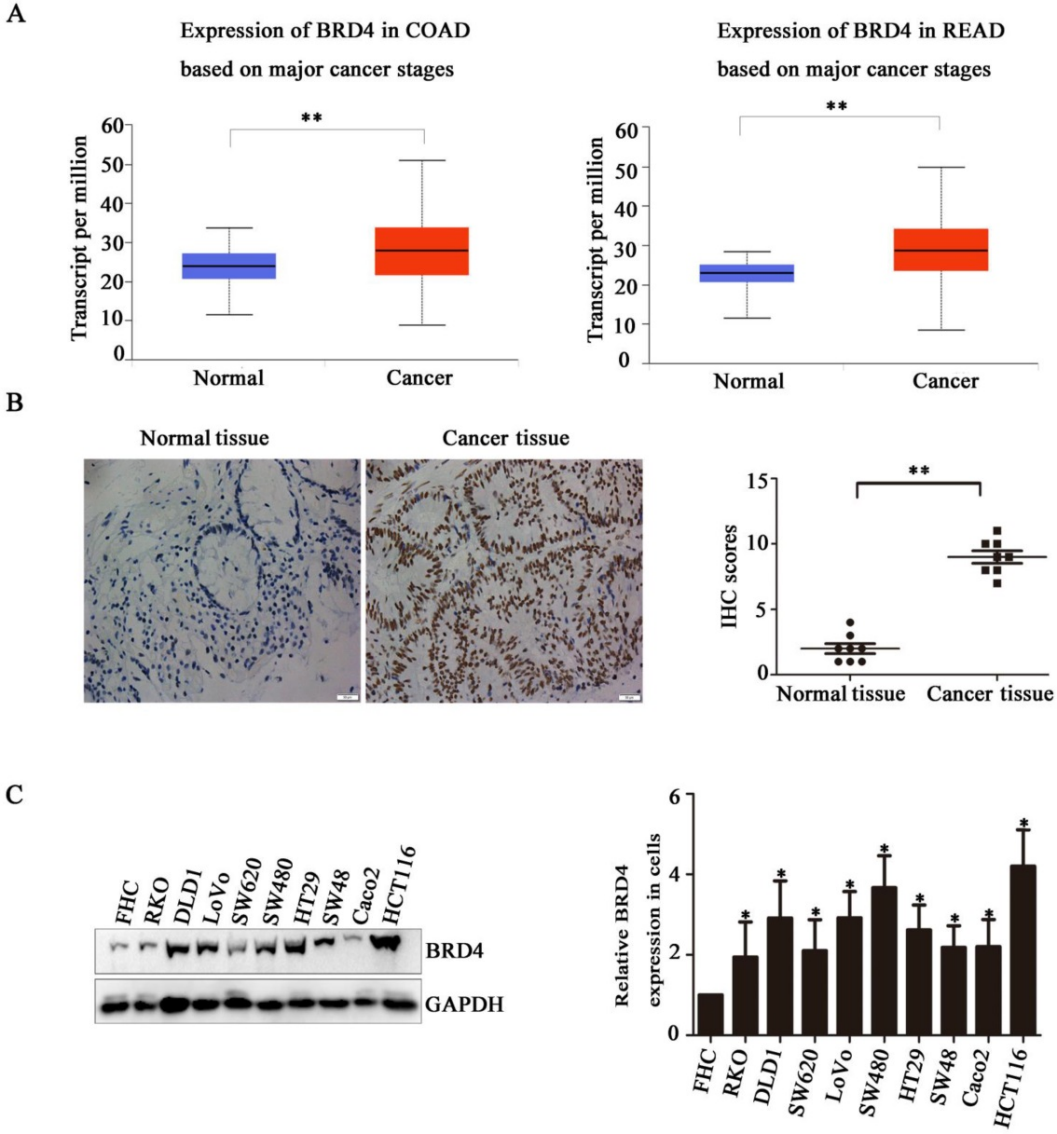
Bromodomain-containing protein 4 (*BRD4*) gene is highly expressed in colon cancer. A) Relative expression level of BRD4 in tumor tissue (both colon and rectal cancer compared with normal tissue. B) Representative images of immunohistochemical staining of BRD4 in normal colonic epithelial tissues and colon cancer epithelial tissue. Magnification, 400×. C) Western blot analysis of expression of BRD4 in human colon cancer cell lines (HCT116, LoVo, RKO, Caco2, HT29, Colo205, SW48, SW480, SW620, and DLD1 cells) and the human normal colonic FHC epithelial cell line. D) Densitometric analysis of blots for 10 cell lines analyzed using Student's *t*-tests (***p<0.001,**p<0.01, *p<0.05)

Based on the indicated role of BRD4 in malignant disease, we analysed the expression of BRD4 in colorectal cancer patient using TCGA database, as showed in (Figure 1A), compared with normal tissues, the level of BRD4 were significantly increased in colorectal cancer tissues (p<0.001). Then, we performed IHC staining in colorectal cancer patients, which also confirmed the higher expression of BRD4 in cancer tissues (Figure 1C, P<0.001). Furthermore, we examined the expression of BRD4 in 9 human colorectal cancer cell lines(HCT116, LoVo, RKO, Caco2, HT29, SW48, SW480, SW620, and DLD1 cells) and normal colorectal epithelial cell FHC, which also indicated the same result that BRD4 was overexpressed in human colorectal cancer cells (Figure 1.D).

### BRD4 Inhibition Suppresses Human Colorectal Cancer Cell Proliferation

**Figure 2:**
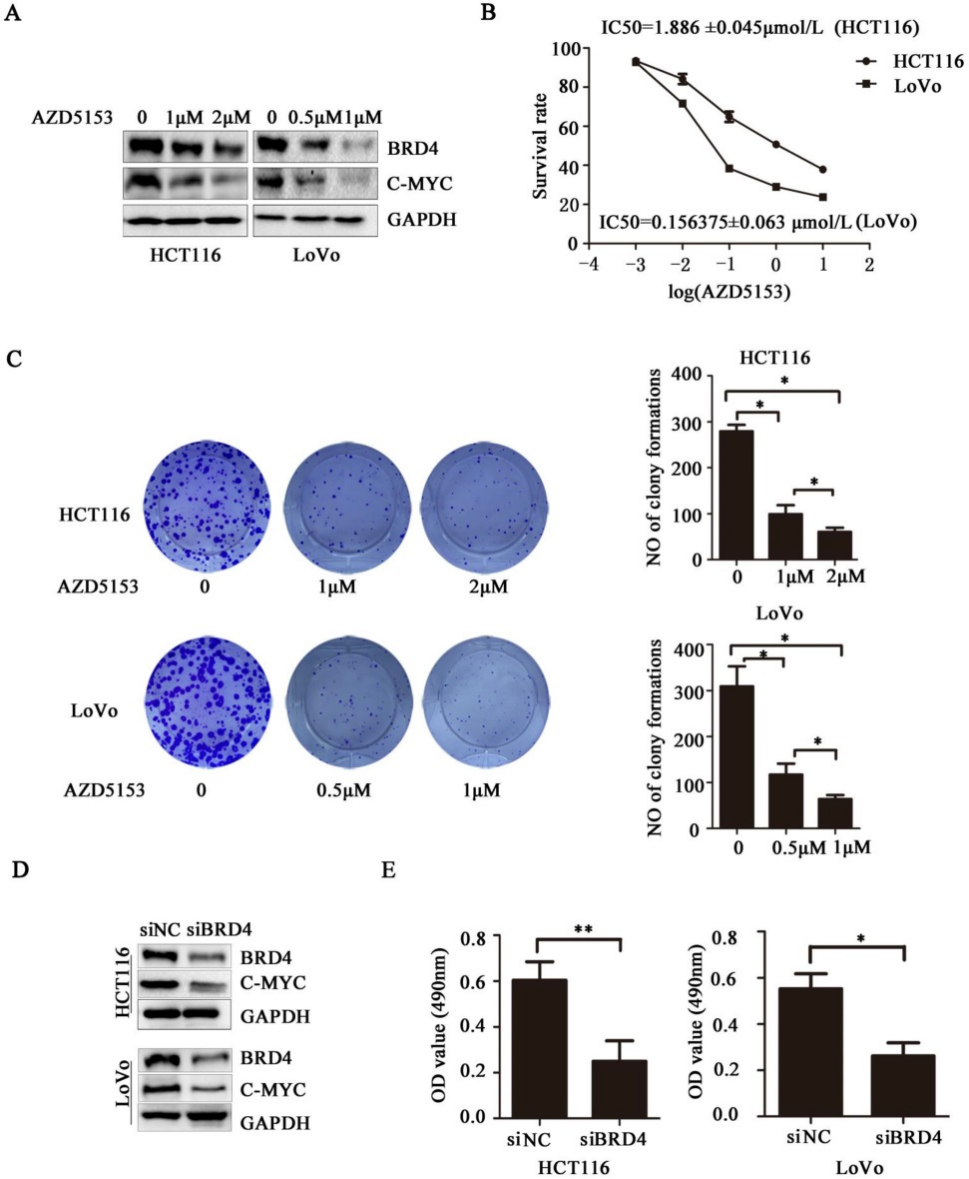
** BRD4 Inhibition Suppresses Human Colorectal Cancer Cell Proliferation**. A) Western blot analysis of expression of BRD4 and c-*Myc* after treatment with AZD5153 in HCT116 and LoVo cells. B) Cell survival and proliferation following treatment of HCT116 and LoVo colon cancer cells the BRD4 small molecule inhibitor, AZD5153, for 72h.TheMTTassay was used to assess survival and proliferation. C) Clonogenic assay following 10-day treatment with AZD5153 and quantification of colony counts. D) Western blot analysis of expression of BRD4 and c-Myc after knockdown of BRD4 in HCT116 and LoVo cells. E) HCT116 and LoVo cells tested using MTT assay. The data were analyzed using Student's *t*-tests(***p<0.001,**p<0.01, *p<0.05).

Next, we evaluated the therapeutic potential of targeting BRD4 in colorectal cancer cells, western blot indicated AZD5153 inhibited BRD4 expression and its important transcriptional regulator *c-Myc* (Figure 2.A), also it could suppress cell proliferation with dose dependent manner in human colorectal cancer cell lines HCT116 and Lovo (Figure 2.B), and the results of colonogenic assay also confirmed the potent anticancer effect of AZD5153 (Figure 2.C). To further confirm the therapeutic value of targeting BRD4, we inhibited BRD4 using specific siRNA, which also indicated BRD4 inhibition also down-regulated the* c-Myc* expression (Figure 2.D). Besides, BRD4 inhibition also reduced the cell viability in HCT116 and LoVo cells (Figure 2.E). To sum up, our data revealed that BRD4 might be a valuable therapeutic target in colorectal cancer cells.

### AZD5153 Induces Apoptosis of Human Colorectal Cancer Cells

**Figure 3:**
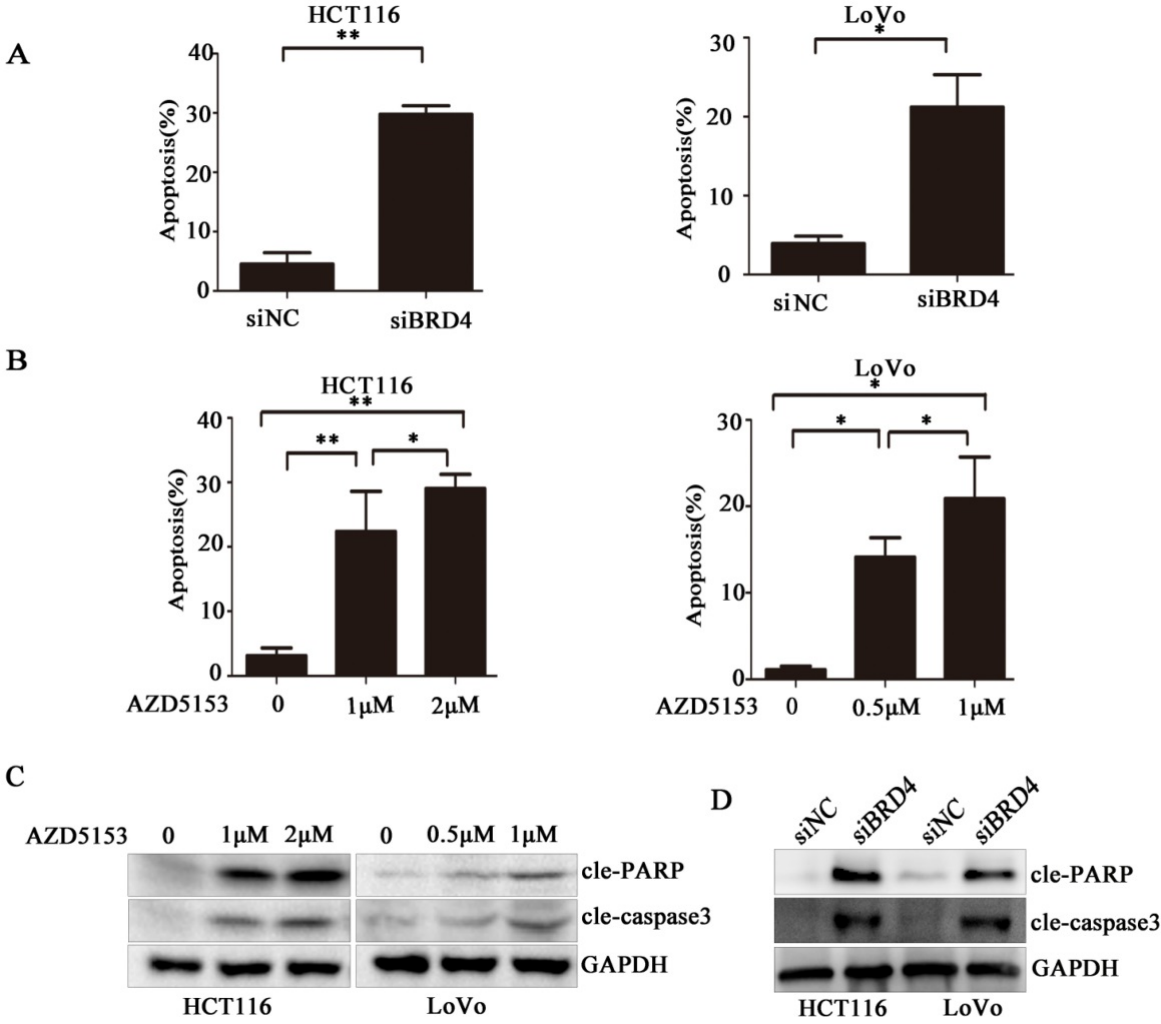
AZD5153 induces apoptosis of human colon cancer cells. A) Flow cytometric analysis of HCT116 and LoVo cells with siRNA-mediated knockdown of BRD4 after 48 h,costaining with propidium iodide (PI) and Annexin V. Percentage of early and late apoptotic cells measured using Annexin V staining of HCT116 and LoVo cells is shown. B) Apoptosis of HCT116 and LoVo cells treated with AZD5153 for 48 h. Percentage of early and late apoptotic cells was measured using Annexin V staining of HCT116 and LoVo cells. C &D)Western blot analysis of expression of cleaved-poly(ADP-ribose) polymerase (PARP) and cleaved-caspase-3 in HCT116 and LoVo cells treated with vehicle (0.01% dimethyl sulfoxide (DMSO)), AZD5153,or BRD4 siRNA. The data were analyzed using Student's *t*-tests (***p<0.001,**p<0.01, *p<0.05).

To clarify the biological activity of AZD5153, we used flow cytometry to detect changes in apoptosis of cells exposed to AZD5153, and examined changes in apoptosis after knocking down the *BRD4* gene with siRNA. After 48 h, the experiment showed clearly that drug treatment or gene knockdown affected the level of apoptosis in the cell lines (Fig. 3 A & B). Moreover, this apoptosis was drug concentration-dependent, and the results were statistically significant. Lastly, we studied the molecule changes of AZD5153treatment and BRD4 knockdown, and found that cleaved-caspase3 and cleaved PARP were significantly increased in AZD553 treated and BRD4 knockdown cells (Fig. 3 C & D).

### AZD5153 Impairs G2M Cell Cycle Checkpoint and Sensitizes the anticancer effect of PARP inhibitor in Colorectal Cancer Cells

**Figure 4:**
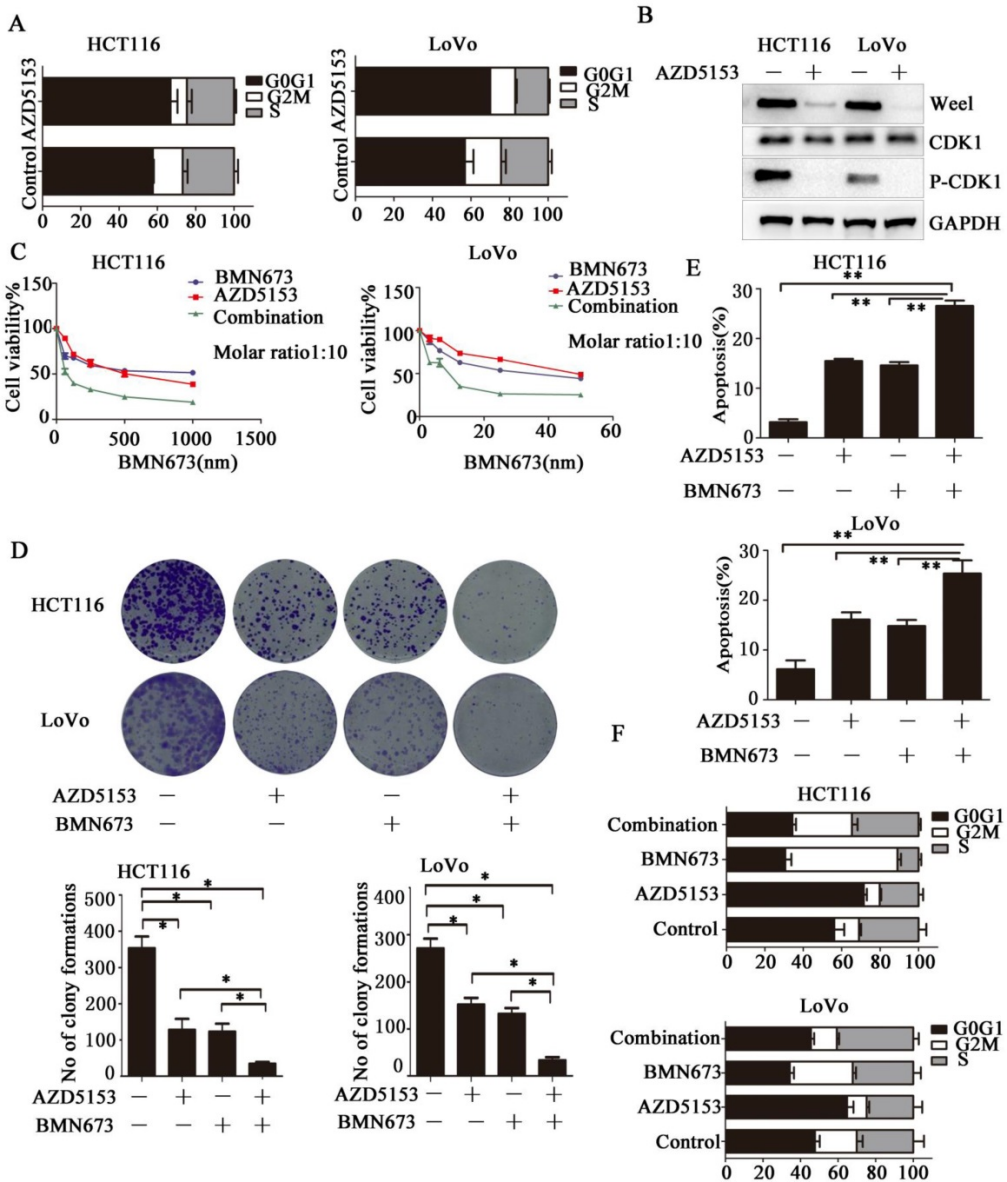
AZD5153 Impairs G2M Cell Cycle Checkpoint and Sensitizes the anticancer effect of PARP inhibitor in Colorectal Cancer Cells. A) Significant G0/G1 phase arrest in AZD5153-treated HCT116 and LoVo cells. B) AZD5153 inhibited the expression of Wee1 and phosphorylation of CDK1 in HCT116 and Love cells. C& D) MTT and clonogenic assay. AZD5153 and BMN673 combination significantly suppressed the proliferation of HCT116 and LoVo cells. E) Flow cytometric analysis of cells treated with AZD5153 and BMN673 shows that combined AZD5153 and BMN673 significantly Promoted the apoptotic of HCT116 and LoVo cells. F) Flow cytometric cell cycle analyses following the indicated treatment for 48 h in HCT116 and LoVo cells show that AZD5153 impaired the G2M checkpoint and allowed more entry into mitosis with DNA damage induced by BMN673.The data were analyzed using Student's *t*-test (***p<0.001,**p<0.01, *p<0.05).

**Figure 5:**
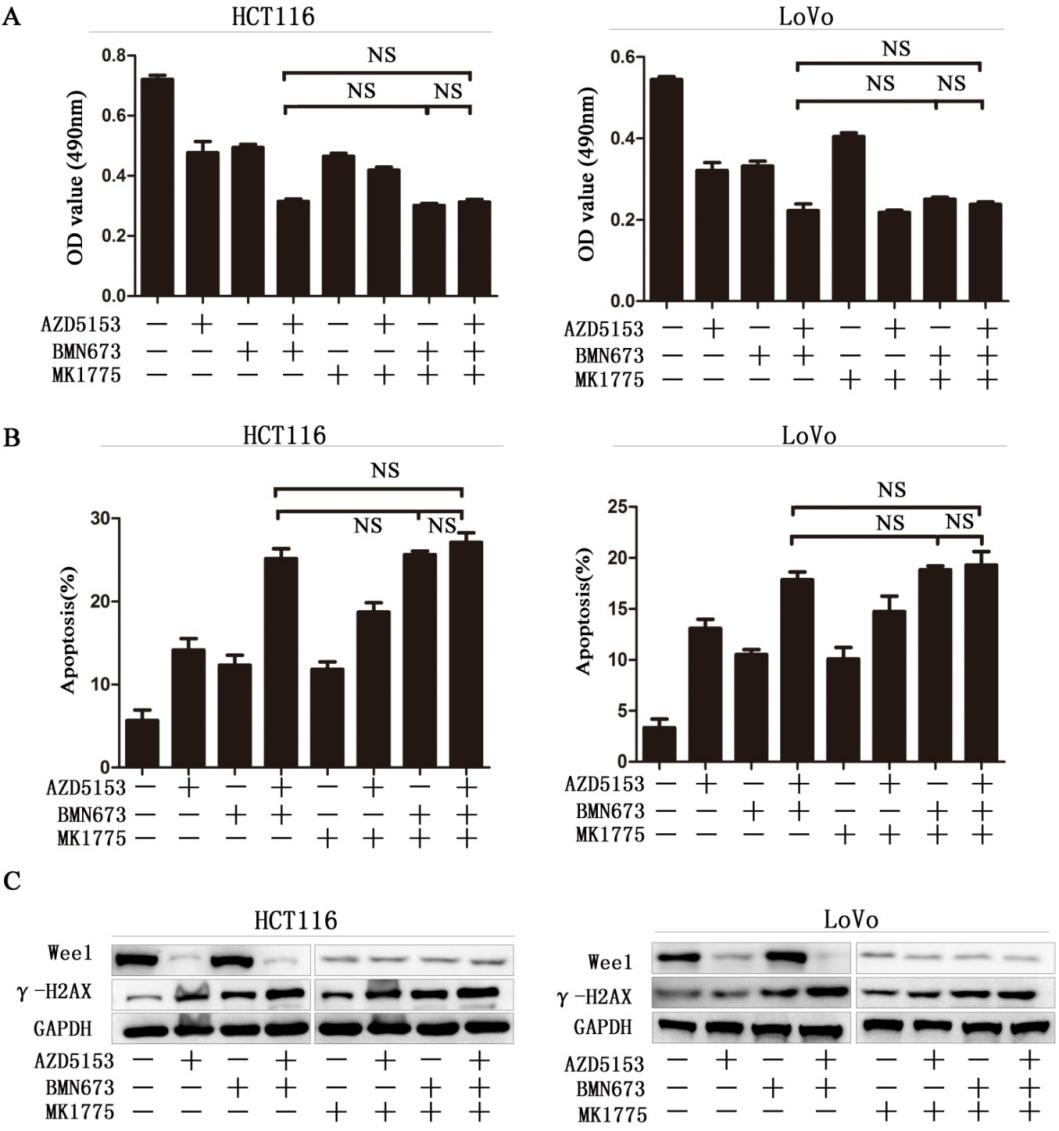
** Wee1 inhibition reverses the sensitization effect of AZD5153 for PARP inhibitor in colorectal cancer cells.**A)Cell survival and proliferation following treatment of HCT116 and LoVo colon cancer cells with AZD5153、BMN673and MK1775(individually and in combination), for 72h.TheMTT assay was used to assess survival and proliferation.B) Apoptosis of HCT116 and LoVo cells treated AZD5153、BMN673and MK1775(individually and in combination) for 48 h. Percentage of early and late apoptotic cells was measured ofHCT116 and LoVo cells.C) Western blot analysis of expression of Wee1 and γ-H2AX after treatment with AZD5153、BMN673and MK1775(individually and in combination).The data were analyzed using Student's *t*-test. NS:*P*≥0.05.

Then we investigated the potential mechanism involved in the anticancer effect of AZD5153 in colorectal cancer, we determined the effect of AZD5153 treatment on cell cycle distribution, and found significant G0/G1 phase arrest in AZD5153-treated cells (Fig. 4A).This indicated a potential role of AZD5153 in cell cycle progression. Moreover, AZD5153 inhibited the expression of Wee1 and phosphorylation of CDK1, which were recognized as key factors in the G2M checkpoint in HCT116 and LoVo cells (Fig. 5B). These results indicated that AZD5153 could sensitize cells to the anticancer effects of DNA damage-related drugs, especially these reagents could induce G2M arrest. PARP inhibitor is the most promising anticancer drugs, but in CRC, PARP inhibitor monotherapy showed limited effect. PARP inhibitor combined with these drugs impaired G2M checkpoint were reported to have potent therapeutic effect^29^, ^30^. Thus, we tested the therapeutic effect of AZD5153 and the PARP inhibitorBMN673in colorectal cancer cells. As shown in (Fig. 4 C & D), AZD5153 and BMN673combination significantly suppressed the proliferation of HCT116 and LoVo cells. Moreover, this combination induced more apoptosis than either monotherapy in these two cell lines (Fig. 4E).PI staining showed that AZD5153 impaired G2M checkpoint and pushed more HCT116 and Lovo cells with DNA damage induced by BMN673 into mitosis (Fig. 4F). Therefore, we provided relevant data that AZD5153 inhibited the expression of Wee1 and impaired the G2M checkpoint, thus sensitized the anticancer effect of BMN673 in colorectal cancer cells.

### Wee1 inhibition reverses the sensitization effect of AZD5153 for PARP inhibitor in colorectal cancer cells

To further confirm Wee1 was involved in the anticancer effect of AZD5153 and BMN673 combination in colorectal cancer. First, we checked the cell viability of combination therapy groups in Wee1 knockdown colorectal cancer cells, contrasted to wild type cells, combination treatment showed limited effect than monotherapy groups in Wee1 knockdown cells (Fig. 5A). Then the data of apoptosis induction also showed that Wee1 knockdown reversed the sensitization effect of AZD5153 on PARP inhibitor (Fig. 5B). Besides, western blot further revealed that the DNA damage marker had limited changed in combination that monotherapy groups in HCT116 and LoVo cells (Fig. 5C). This data provided evidence that AZD5153 sensitized the anticancer effect of PARP inhibitor via Wee1 inhibition.

### AZD5153 Improves the Therapeutic Efficacy of BMN673 in Colorectal Cancer Cells *In vivo*

**Figure 6:**
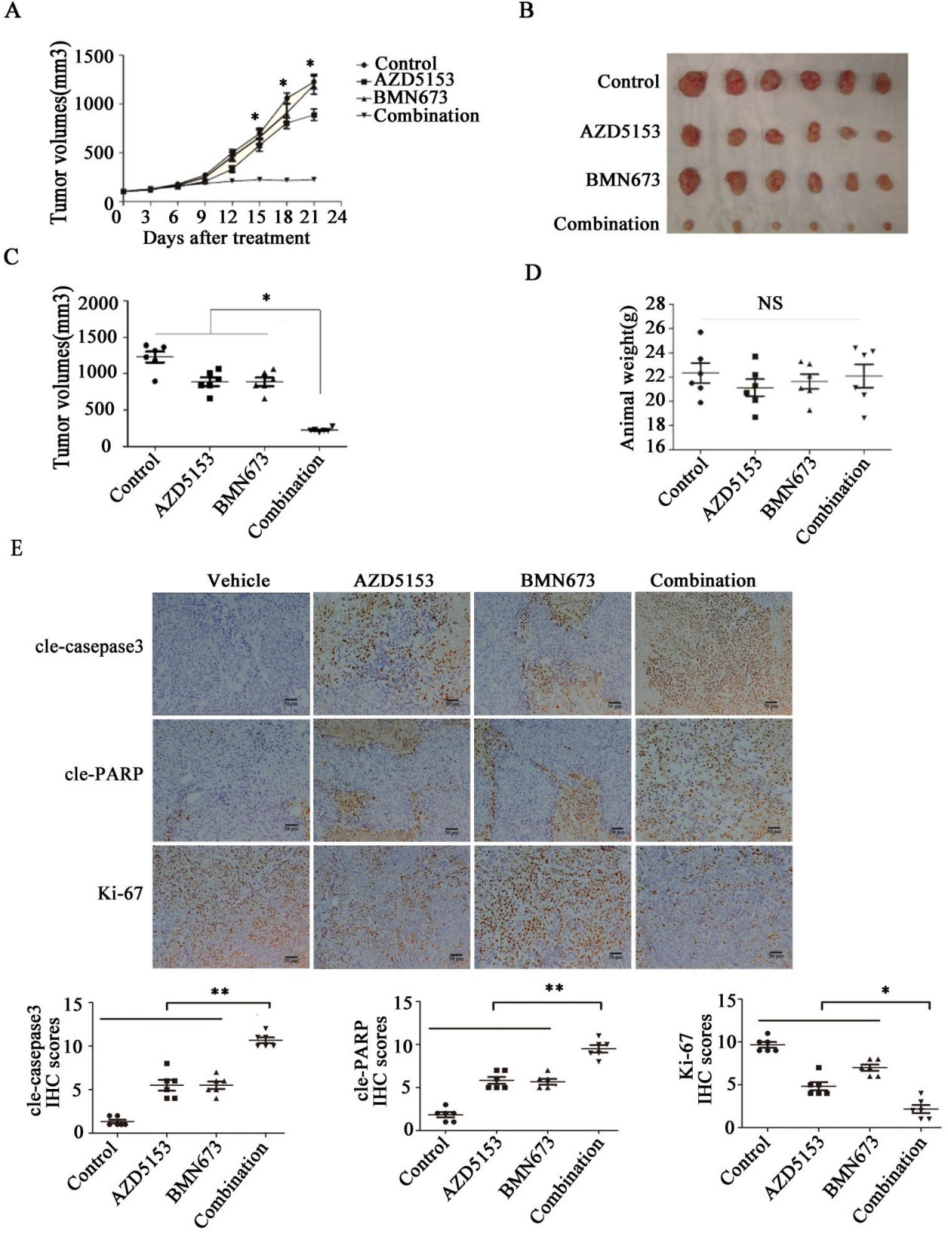
** AZD5153 improves therapeutic efficacy of the PARP inhibitor, BMN673, in colorectal cancer cells *in vivo***. Male and female nude mice (n = 6 per group) were orthotopically implanted with HCT116 tumor xenografts and given treatment with a vehicle control, AZD5153 (5 mg/kg), BMN673 (0.33mg/kg), or a combination of these two agents via oral gavage for 3 weeks. A) Resulting tumor volumes measured on the indicated days of treatment. B&C) Representative photograph of orthotopically implanted tumors in each mouse group at the time of study termination (day 21). Scale bar = 1 cm. Results are shown as means ± standard error of the mean. Two-way analysis of variance was used to determine the statistical significance of differences among groups. *p<0.05. D) Body-weight versus time curve for mice with xenografts. Two-way analysis of variance was used to determine the statistical significance of differences among groups. n.s, not significant. E) Representative images of H&E, Ki-67, cleaved-poly(ADP-ribose) polymerase (PARP), and cleaved-caspase-3 immunohistochemistry. Magnification, 200×.Two-way analysis of variance was used to determine the statistical significance of differences among groups. *p<0.05,**p<0.01,NS:*P*≥0.05.

Lastly, we generated a nude mice xenograft model to verify the anticancer effect of co-treatment with AZD5153 and BMN673 *in vivo*. After continuously treating with the indicated drugs for 3 weeks, the average tumor volume in the combination group was significantly lower than those in the monotherapy groups (Fig. 6A, B&C). More importantly, there was no significant difference among the mouse weights in each group, which indicated that this drug combination was tolerable in the animal model (Fig. 6D). Furthermore, the immunohistochemical analysis of the tumor tissue confirmed the effect of AZD5153 and BMN673 on tumor cell growth and induction of apoptosis (Fig. 6E).Therefore, our data demonstrated that the combination of AZD5153 and BMN673 had potent anticancer effects *in vivo*.

## Discussion

The BET family members consist of four proteins, BRD2, BRD3, BRD4, and BRDT.BRDT is only expressed in the testis, whereas the other three members are widely expressed in tissues and cells^10^, ^31^. BRD2 is a member of the “Beta” subfamily of the bromo structure-containing proteins and was the first BET gene identified in mammals. Typical conserved bromodomains are found in many eukaryotic chromosomal remodeling and transcription activator or inhibitor proteins, confirming that BRD2 selectively binds lysine side chains on histones H3 and H4. The two sequence structures of BRD2 suggest that it may have both activating and inhibiting functions. BRD3 and BRD2 are highly similar, and both bind acetylated histones and initiate gene transcription in the absence of the facilitates chromatin transcription (FACT) complex. However, their functions are not identical. In addition to histone binding, BRD3 molecules bind to several transcription factors. BRD3 has attracted much attention in immunoregulation. As mentioned before, BRD2 may have both activation and inhibition functions, which was also confirmed by the bidirectional effect of JQ1, an inhibitor of the BRD4/BRD2 target, in colon cancer. Therefore, a single target therapy against BRD4 is considered superior to BRD4/BRD2 dual-targeting 22, 23, 32, 33.

BRD4 is a member of BET family which contains two bromodomains and a hyperterminal structure that binds via the bromodomain throughout the cell cycle. BRD4 also causes the activation of RNA polymerase II, which promotes the expression of growth-promoting genes such as* c-Myc*^13^. The *c-Myc* gene was found to be overexpressed, translocated, or activated in various hematological tumors and was once considered a tumor target that could not be adjusted by drugs. Small-molecule inhibitors of BET show potent inhibition of *c-Myc* and its downstream target molecules, causing proliferation inhibition^3^, ^31^, ^34^. In recent years, studies have shown the presence of many epigenetic abnormalities in colon cancer, and unlike genetic mutations, epigenetic changes are reversible 2. Therefore, the development of drugs targeting epigenetic mechanisms has provided new research ideas for the treatment of CRC. BRD4 is inextricably linked with epigenetics 3, 35. Herein, we evaluated the characteristics of the novel BRD4 inhibitor, AZD5153. The bivalent binding mode of AZD5153 makes it different from the previously described BRD4 inhibitors. AZD5153 mechanically and simultaneously connects to two BRD4 bromodomains, which allows a low dose to efficiently displace BRD4 from chromatin. This unique biophysical property translates into *in vitro* and *in vivo* enhancements of its pharmacological activity. AZD5153 exhibits potent anticancer effects in a variety of hematopoietic cancers (such as acute myeloid leukemia), malignant peripheral nerve sheath tumors, and medulloblastomas^24^-^27^. However, some solid tumors such as lung, breast, and cervical cancers are less sensitive to AZD5153 [36][35][34]. A previous study reported that AZD5153 can inhibit cancer cell proliferation and promote apoptosis in thyroid carcinoma cells, thereby confirming its potential therapeutic effect 25. Nevertheless, there is a lack of convincing research on the effects of AZD5153 on CRC. Consequently, we suggest that AZD5153 inhibits the survival and proliferation of colon cancer cells at micromolar concentrations and promotes their apoptosis. It is ineffective against, and not cytotoxic to normal colonic epithelial cells.

In our study, we reported that the BRD4 inhibitor, AZD5153, potently suppressed the proliferation and induced apoptosis of colorectal cancer cells *in vitro* and *in vivo*. A series of drugs showed promising preclinical anticancer effects in animal models. However, the clinical trials failed for several reasons, and toxicity was considered a major obstacle for their clinical application. Based on this, it is reasonable to develop rational combination therapies. We found that AZD5153 inhibited the expression of Wee1 and impaired the G2M checkpoint; this provided an opportunity to combine AZD5153 and DNA damage-related PARP inhibitors. Our data showed that AZD5153 improved the anticancer therapeutic efficacy of the PARP inhibitor, BMN673, *in vitro* and *in vivo*, and it showed limited toxicity in our nude mouse model.

## Conclusion

In summary, AZD5153 is a novel BET bromodomain inhibitor with a bivalent binding mode. The unique biophysical properties of AZD5153 offer potency and pharmacological advantages over traditional monovalent BET bromodomain inhibitors. Besides, AZD5153 significantly improved the efficacy of the PARP inhibitor, BMN673, *in vitro* and *in vivo*. Based on the potent *in vivo* antitumor activity of AZD5153, BRD4 is suggested to be a promising target for the treatment of CRC.

## Abbreviations

CRC: colorectalcancer
FDA: drug Administration
EGFR: epidermal growth factor receptor
VEGF: vascular endothelial growth factor
BET: bromodomain and extra-terminal domain
BRD4: bromodomain-containing protein 4
HCC: hepatocellularcarcinoma
MTT: 3-(4,5-Dimethylthiazol-2-yl)-2,5-diphenyltetrazolium Bromide
PMSF: phenylmethanesulfonyl fluoride
RIPA: radioimmunoprecipitation assay
BCA: bicinchoninic acid

## References

[1] Potter MB (2013). Strategies and resources to address colorectal cancer screening rates and disparities in the United States and globally. Annual review of public health.

[2] Marino D, Leone F, D'Avanzo F, Ribero D, Capussotti L, Aglietta M (2014). Potentially resectable metastatic colorectal cancer: an individualized approach to conversion therapy. Critical reviews in oncology/hematology.

[3] Togel L, Nightingale R, Chueh AC, Jayachandran A, Tran H, Phesse T (2016). Dual Targeting of Bromodomain and Extraterminal Domain Proteins, and WNT or MAPK Signaling, Inhibits c-MYC Expression and Proliferation of Colorectal Cancer Cells. Molecular cancer therapeutics.

[4] Di Franco S, Todaro M, Dieli F, Stassi G (2014). Colorectal cancer defeating? Challenge accepted!. Mol Aspects Med.

[5] Porcellini E, Laprovitera N, Riefolo M, Ravaioli M, Garajova I, Ferracin M (2018). Epigenetic and epitranscriptomic changes in colorectal cancer: Diagnostic, prognostic, and treatment implications. Cancer letters.

[6] Bhattacharya R, Fan F, Wang R, Ye X, Xia L, Boulbes D (2017). Intracrine VEGF signalling mediates colorectal cancer cell migration and invasion. British journal of cancer.

[7] Bhattacharya R, Ye XC, Wang R, Ling X, McManus M, Fan F (2016). Intracrine VEGF Signaling Mediates the Activity of Prosurvival Pathways in Human Colorectal Cancer Cells. Cancer research.

[8] Canavese M, Ngo DT, Maddern GJ, Hardingham JE, Price TJ, Hauben E (2017). Biology and therapeutic implications of VEGF-A splice isoforms and single-nucleotide polymorphisms in colorectal cancer. International journal of cancer.

[9] Koustas E, Karamouzis MV, Mihailidou C, Schizas D, Papavassiliou AG (2017). Co-targeting of EGFR and autophagy signaling is an emerging treatment strategy in metastatic colorectal cancer. Cancer letters.

[10] Andrieu GP, Denis GV (2018). BET Proteins Exhibit Transcriptional and Functional Opposition in the Epithelial-to-Mesenchymal Transition. Molecular cancer research: MCR.

[11] French CA (2016). Small-Molecule Targeting of BET Proteins in Cancer. Advances in cancer research.

[12] Jung M, Gelato KA, Fernandez-Montalvan A, Siegel S, Haendler B (2015). Targeting BET bromodomains for cancer treatment. Epigenomics.

[13] Ba M, Long H, Yan Z, Wang S, Wu Y, Tu Y (2018). BRD4 promotes gastric cancer progression through the transcriptional and epigenetic regulation of c-MYC. Journal of cellular biochemistry.

[14] Belkina AC, Nikolajczyk BS, Denis GV (2013). BET protein function is required for inflammation: Brd2 genetic disruption and BET inhibitor JQ1 impair mouse macrophage inflammatory responses. Journal of immunology.

[15] Holscher AS, Schulz WA, Pinkerneil M, Niegisch G, Hoffmann MJ (2018). Combined inhibition of BET proteins and class I HDACs synergistically induces apoptosis in urothelial carcinoma cell lines. Clinical epigenetics.

[16] Singh AR, Joshi S, Burgoyne AM, Sicklick JK, Ikeda S, Kono Y (2016). Single Agent and Synergistic Activity of the "First-in-Class" Dual PI3K/BRD4 Inhibitor SF1126 with Sorafenib in Hepatocellular Carcinoma. Molecular cancer therapeutics.

[17] Xiang T, Bai JY, She C, Yu DJ, Zhou XZ, Zhao TL (2018). Bromodomain protein BRD4 promotes cell proliferation in skin squamous cell carcinoma. Cellular signalling.

[18] Xiang Q, Zhang Y, Li J, Xue X, Wang C, Song M (2018). Y08060: A Selective BET Inhibitor for Treatment of Prostate Cancer. ACS medicinal chemistry letters.

[19] Wilson AJ, Stubbs M, Liu P, Ruggeri B, Khabele D (2018). The BET inhibitor INCB054329 reduces homologous recombination efficiency and augments PARP inhibitor activity in ovarian cancer. Gynecologic oncology.

[20] Wang L, Wu X, Wang R, Yang C, Li Z, Wang C (2017). BRD4 inhibition suppresses cell growth, migration and invasion of salivary adenoid cystic carcinoma. Biological research.

[21] Dawson MA, Gudgin EJ, Horton SJ, Giotopoulos G, Meduri E, Robson S (2014). Recurrent mutations, including NPM1c, activate a BRD4-dependent core transcriptional program in acute myeloid leukemia. Leukemia.

[22] Kamijo H, Sugaya M, Takahashi N, Oka T, Miyagaki T, Asano Y (2017). BET bromodomain inhibitor JQ1 decreases CD30 and CCR4 expression and proliferation of cutaneous T-cell lymphoma cell lines. Archives of dermatological research.

[23] Wroblewski M, Scheller-Wendorff M, Udonta F, Bauer R, Schlichting J, Zhao L (2018). BET-inhibition by JQ1 promotes proliferation and self-renewal capacity of hematopoietic stem cells. Haematologica.

[24] Bradbury RH, Callis R, Carr GR, Chen H, Clark E, Feron L (2016). Optimization of a Series of Bivalent Triazolopyridazine Based Bromodomain and Extraterminal Inhibitors: The Discovery of (3R)-4-[2-[4-[1-(3-Methoxy-[1,2,4]triazolo[4,3-b]pyridazin-6-yl)-4-piperidyl]phenoxy]ethyl]-1,3-dimethyl-piperazin-2-one (AZD5153). Journal of medicinal chemistry.

[25] Rhyasen GW, Hattersley MM, Yao Y, Dulak A, Wang W, Petteruti P (2016). AZD5153: A Novel Bivalent BET Bromodomain Inhibitor Highly Active against Hematologic Malignancies. Molecular cancer therapeutics.

[26] Xu K, Chen D, Qian D, Zhang S, Zhang Y, Guo S (2018). AZD5153, a novel BRD4 inhibitor, suppresses human thyroid carcinoma cell growth *in vitro* and *in vivo*. Biochemical and biophysical research communications.

[27] Shen G, Chen J, Zhou Y, Wang Z, Ma Z, Xu C (2018). AZD5153 Inhibits Prostate Cancer Cell Growth *in vitro* and *in vivo*. Cellular physiology and biochemistry: international journal of experimental cellular physiology, biochemistry, and pharmacology.

[28] Collins TA, Hattersley MM, Yates J, Clark E, Mondal M, Mettetal JT (2017). Translational Modeling of Drug-Induced Myelosuppression and Effect of Pretreatment Myelosuppression for AZD5153, a Selective BRD4 Inhibitor. CPT: pharmacometrics & systems pharmacology.

[29] Andrei AZ, Hall A, Smith AL, Bascunana C, Malina A, Connor A (2015). Increased *in vitro* and *in vivo* sensitivity of BRCA2-associated pancreatic cancer to the poly(ADP-ribose) polymerase-1/2 inhibitor BMN 673. Cancer letters.

[30] Tempka D, Tokarz P, Chmielewska K, Kluska M, Pietrzak J, Rygielska Z (2018). Downregulation of PARP1 transcription by CDK4/6 inhibitors sensitizes human lung cancer cells to anticancer drug-induced death by impairing OGG1-dependent base excision repair. Redox biology.

[31] Devaiah BN, Lewis BA, Cherman N, Hewitt MC, Albrecht BK, Robey PG (2012). BRD4 is an atypical kinase that phosphorylates serine2 of the RNA polymerase II carboxy-terminal domain. Proceedings of the National Academy of Sciences of the United States of America.

[32] Ozer HG, El-Gamal D, Powell B, Hing ZA, Blachly JS, Harrington B (2018). BRD4 Profiling Identifies Critical Chronic Lymphocytic Leukemia Oncogenic Circuits and Reveals Sensitivity to PLX51107, a Novel Structurally Distinct BET Inhibitor. Cancer discovery.

[33] Stathis A, Bertoni F (2018). BET Proteins as Targets for Anticancer Treatment. Cancer discovery.

[34] Di Micco R, Fontanals-Cirera B, Low V, Ntziachristos P, Yuen SK, Lovell CD (2014). Control of embryonic stem cell identity by BRD4-dependent transcriptional elongation of super-enhancer-associated pluripotency genes. Cell reports.

[35] Wu Z, Hu Z, Han X, Li Z, Zhu Q, Wang Y (2017). The BET-Bromodomain Inhibitor JQ1 synergized ABT-263 against colorectal cancer cells through suppressing c-Myc-induced miR-1271-5p expression. Biomedicine & pharmacotherapy = Biomedecine & pharmacotherapie.

[36] Robert H (2018). Bradbury1 RC, Gregory R. Carr1, Huawei Chen2, Edwin Clark2, Lyman Feron1, Steve Glossop1 MAG, Maureen Hattersley2, Chris Jones1, Scott G. Lamont1, Gilles, Ouvry3 AP, Joe Patel2, Alfred A. Rabow1, Craig A. Roberts1, Stephen Stokes1, Natalie, Stratton1 GEW, Lara Ward1, David Whalley1, David Whittaker1, Gail Wrigley1 and, Michael J. Waring1.

