# BRD4 Inhibitor AZD5153 Suppresses the Proliferation of Colorectal Cancer Cells and Sensitizes the Anticancer Effect of PARP Inhibitor: Erratum

**DOI:** 10.7150/ijbs.73868

**Published:** 2022-05-27

**Authors:** Peng Zhang, Ruidong Li, Hua Xiao, Weizhen Liu, Xiangyu Zeng, Genchen Xie, Wenchang Yang, Liang Shi, Yuping Yin, Kaixiong Tao

**Affiliations:** 1 Department of Gastrointestinal Surgery, Union Hospital, Tongji Medical College, Huazhong University of Science and Technology, Wuhan 430022, China;; 2 Department of Gastroduodenal and Pancreatic Surgery, Hunan Cancer Hospital and the Affiliated Cancer Hospital of Xiangya School of Medicine, Central South University, No. 283 Tongzipo Road, Changsha, Hunan Province 410013, China.

Due to improper processing of Western Blot images, there were four errors in our paper. We reviewed all the raw data in detail and found figure 1C and Figure 3C misused the GAPDH band. The Wee1 bands of LoVo cells in Figure 5C were incorrectly placed, and the GAPDH bands of LoVo cells in Figure 5C were incorrectly placed. We regret that we did not catch these errors before publication. Here we showed the corrected Figures 1C, Figures 3C and 5C with side by side comparison of the previous figures. The data analyses and conclusion remain unchanged.

## Figures and Tables

**Figure 1:**
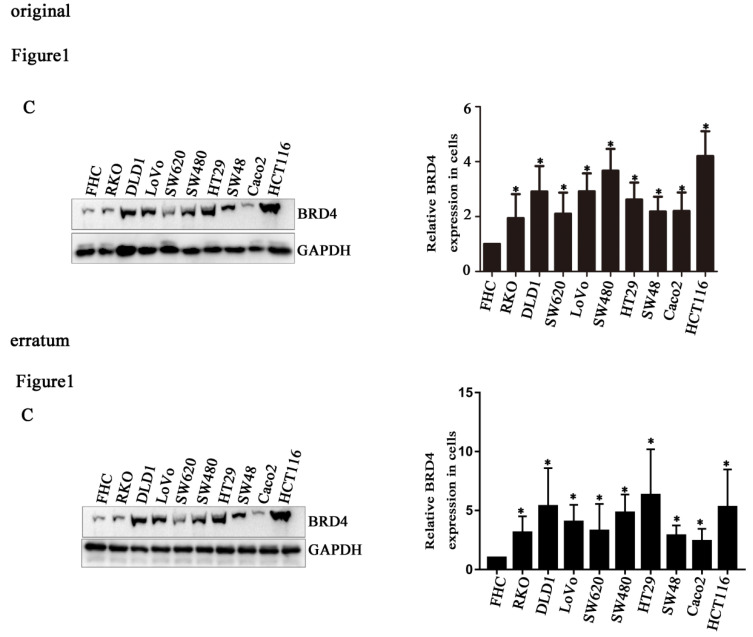
C. Corrected figure.

**Figure 3:**
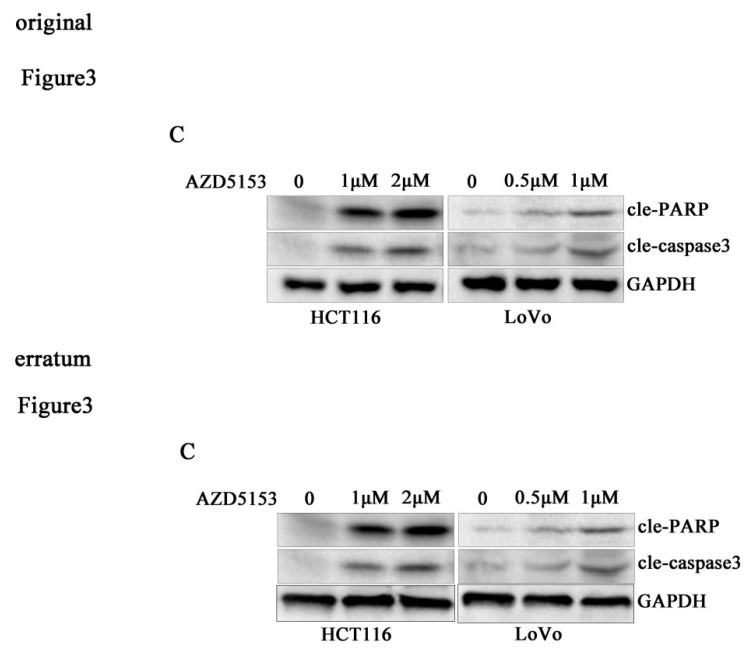
C. Corrected figure.

**Figure 5:**
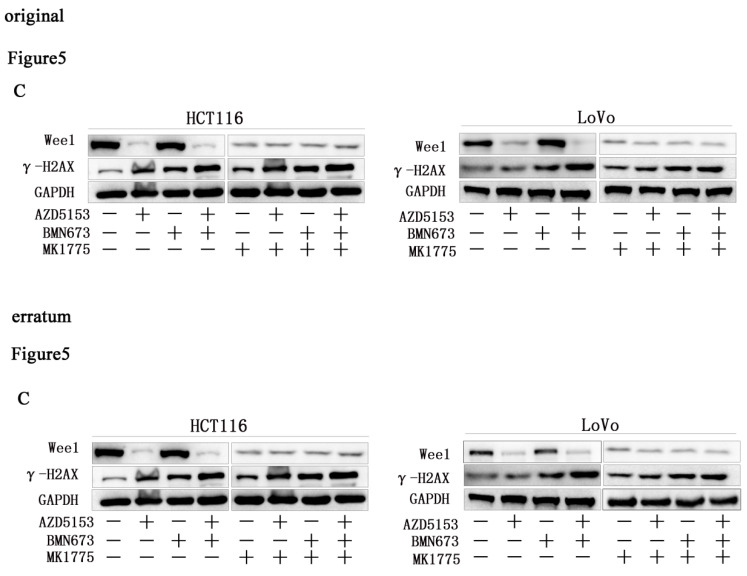
C. Corrected figure.

